# Supplementary material for: Patterns of testing in the extensive Danish national SARS-CoV-2 test set-up
Source: PLoS One. 2023 Jul 25;18(7):e0281972. doi: 10.1371/journal.pone.0281972 (PMC10368237; doi:10.1371/journal.pone.0281972)
Supplement: Attachment — Submitted filename: Response to Reviewers.docx [file pone.0281972.s002.docx]

# **Response to editor and reviewers**

Thank you for taking the time to comment on our manuscript. Your comments have been very helpful to improve the manuscript. The information has been added to the manuscript and a point-by-point response is included below.

## Journal Requirements

1. Please ensure that your manuscript meets PLOS ONE's style requirements, including those for file naming. The PLOS ONE style templates can be found at

<https://journals.plos.org/plosone/s/file?id=wjVg/PLOSOne_formatting_sample_main_body.pdf> and 
<https://journals.plos.org/plosone/s/file?id=ba62/PLOSOne_formatting_sample_title_authors_affiliations.pdf>

**Response:** Thank you for sharing the templates. The manuscript has been revised to meet the style requirements.

2. Thank you for stating the following in the Acknowledgments Section of your manuscript:

"..Many structures in Danish society contributed to establishing and running the test centres, special contributions were given by the Novo Nordic Foundation (Grant number: NNF20SA0063854), Novo Nordisk Denmark (providing staff), McKinsey (sourcing) and the Danish Military (logistical support)."

We note that you have provided funding information that is not currently declared in your Funding Statement. However, funding information should not appear in the Acknowledgments section or other areas of your manuscript. We will only publish funding information present in the Funding Statement section of the online submission form.

Please remove any funding-related text from the manuscript and let us know how you would like to update your Funding Statement. Currently, your Funding Statement reads as follows:

"The authors received no specific funding for this work."

Please include your amended statements within your cover letter; we will change the online submission form on your behalf.

**Response:** Thank you for pointing this out. We have removed the funding-related information from the manuscript. The authors received no funding for the work presented in this paper. However, many structures in Danish society contributed to establishing and running the test centres, special contributions were given by the Novo Nordic Foundation (Grant number: NNF20SA0063854), Novo Nordisk Denmark (providing staff), McKinsey (sourcing) and the Danish Military (logistical support). Can we include this information in the supporting file?

3. In your Data Availability statement, you have not specified where the minimal data set underlying the results described in your manuscript can be found. PLOS defines a study's minimal data set as the underlying data used to reach the conclusions drawn in the manuscript and any additional data required to replicate the reported study findings in their entirety. All PLOS journals require that the minimal data set be made fully available. For more information about our data policy, please see http://journals.plos.org/plosone/s/data-availability.

Upon re-submitting your revised manuscript, please upload your study’s minimal underlying data set as either Supporting Information files or to a stable, public repository and include the relevant URLs, DOIs, or accession numbers within your revised cover letter. For a list of acceptable repositories, please see <http://journals.plos.org/plosone/s/data-availability#loc-recommended-repositories>. Any potentially identifying patient information must be fully anonymized.

Important: If there are ethical or legal restrictions to sharing your data publicly, please explain these restrictions in detail. Please see our guidelines for more information on what we consider unacceptable restrictions to publicly sharing data: <http://journals.plos.org/plosone/s/data-availability#loc-unacceptable-data-access-restrictions>. Note that it is not acceptable for the authors to be the sole named individuals responsible for ensuring data access.

We will update your Data Availability statement to reflect the information you provide in your cover letter.

**Response:** Thank you. The underlying dataset, which consists of more than 120 million data rows, contains person identifiable information concerning most of the Danish population. We are not in a position to share this and we believe that we have provided an explanation for this already. Should we have misunderstood, we’re of course happy to discuss what to do. The text we previously provided reads:
*“The data material used involve information on every person living in Denmark within the study period. Data cannot be shared publicly because they may only be accessed and register-coupled within a secure data analysis environment. However, de-identified participant-level data are available for Institutional Data access to members of the scientific and medical community for non-commercial use only. Applications should be submitted to Forskerservice (https://sundhedsdatastyrelsen.dk/da/forskerservice) at The Danish Health Data Authority, where they will be reviewed on the basis of relevance and scientific merit. Data are available now, with no defined end date.”*

4. Your ethics statement should only appear in the Methods section of your manuscript. If your ethics statement is written in any section besides the Methods, please move it to the Methods section and delete it from any other section. Please ensure that your ethics statement is included in your manuscript, as the ethics statement entered into the online submission form will not be published alongside your manuscript.

**Response:** The ethic statement has been moved to the Methods section and deleted from any other section.

5. Please upload a new copy of Figures 1 and 2 as the detail is not clear. Please follow the link for more information:
<https://blogs.plos.org/plos/2019/06/looking-good-tips-for-creating-your-plos-figures-graphics/>
<https://blogs.plos.org/plos/2019/06/looking-good-tips-for-creating-your-plos-figures-graphics/>

**Response:** Thank you for giving us the opportunity to improve the quality of the figures. The figures have been re-created in R studio instead of excel, which makes it easier to adjust the dimensions and resolution. Please let us know if the new version of the figures appears blurry.

6. Please review your reference list to ensure that it is complete and correct. If you have cited papers that have been retracted, please include the rationale for doing so in the manuscript text, or remove these references and replace them with relevant current references. Any changes to the reference list should be mentioned in the rebuttal letter that accompanies your revised manuscript. If you need to cite a retracted article, indicate the article’s retracted status in the References list and also include a citation and full reference for the retraction notice.

**Response**: We have ensured that the reference list is complete and correct.

Review Comments to the Author

Please use the space provided to explain your answers to the questions above. You may also include additional comments for the author, including concerns about dual publication, research ethics, or publication ethics. (Please upload your review as an attachment if it exceeds 20,000 characters)

**Reviewer #1:** The manuscript titled "Patterns of testing in the extensive Danish national SARS-CoV-2 test set-up" is an interesting study.

I appreciate the authors' topic. The authors clearly defined the research questions in the background section. However, the following comments need to be addressed, and the authors should improve their manuscript in the following aspects:

1. Please improve the quality of the manuscript.

**Response:** We believe that, with the number of changes we’ve made to the manuscript, including the new figure, the overall quality of the manuscript has improved considerably. We hope by this to have responded adequately to this comment by the reviewer.

2. This research is important and useful because it will help improve the COVID-19 national health system in every country. This will make it easier to find out things like who is infected or has no symptoms.

Has a comparable study been done in other countries? I think it will help improve the adoption of health programs in the future.

And on the basis of that, a unique regulation was established. Mention it if the answer is positive.

I am aware that the number of samples required for statistical analysis is enormous, but you could summarize the key points of the data in Table 3 in a graph in order to prevent generalization and scattering and to facilitate content transfer for better understanding.

**Response:** To our knowledge no comparable studies have been done in other countries. We appreciate your suggestion of visualizing the data in Table 3 which we have followed. The results in Table 3 are now shown in Fig 3. We believe this new figure provide a better overview of the results of the analysis. Table 3 has been moved to Supplementary and appears as S3 Table. We decided to keep the table in supplementary because it adds additional information of number of events and person-years.

**Reviewer #2:** Dear authors, overall, the manuscript is well written & presents an excellent results, I don't have any scientific revision, only I have comments about figures quality it should be replaced with a good ones. Best wishes.

**Response:** Thank you so much for your comments. The figures have been re-created in R studio instead of excel, which makes it easier to adjust the dimensions and resolution to ensure high quality.
