# Supplementary material for: Patterns of testing in the extensive Danish national SARS-CoV-2 test set-up
Source: PLoS One. 2023 Jul 25;18(7):e0281972. doi: 10.1371/journal.pone.0281972 (PMC10368237; doi:10.1371/journal.pone.0281972)
Supplement: Attachment — Submitted filename: Response to Reviewers.docx [file pone.0281972.s003.docx]

# **Response to editor and reviewers**

## Journal Requirements

Please review your reference list to ensure that it is complete and correct. If you have cited papers that have been retracted, please include the rationale for doing so in the manuscript text, or remove these references and replace them with relevant current references. Any changes to the reference list should be mentioned in the rebuttal letter that accompanies your revised manuscript. If you need to cite a retracted article, indicate the article’s retracted status in the References list and also include a citation and full reference for the retraction notice.

Response: We have reviewed the reference list to ensure that it is complete and correct.

Review Comments to the Author

1) Could you specify if there were mandatory testing before attending mass gathering events, especially during the first wave

Response: Thank you for your valuable comment. We appreciate your consideration of our findings. We agree that test requirements played an important role in the testing patterns. Throughout the pandemic various restrictions including test requirements were implemented and lifted depending on the development of the pandemic. Our colleagues have done a great work describing the restrictions in Figure 1 in a published study *(Munch PK, Espenhain L, Hansen CH, Krause TG, Ethelberg S. Case-control study of activities associated with SARS-CoV-2 infection in an adult unvaccinated population and overview of societal COVID-19 epidemic counter measures in Denmark. PLoS One. 2022;17(11):e0268849. doi: 10.1371/journal.pone.0268849)*. In our study we briefly mention the restrictions and cite our colleagues study for further information. “During certain periods a negative PCR-test was necessary in order take part in civil life (e.g. access to sport/public institutions, restaurants, public cultural activities or foreign travel) [27]. Further, staff at hospitals, nursing homes and home care have actively been encouraged to be voluntarily tested at their workplace several times a week for prolonged periods. Similarly, school children and students were tested weekly or biweekly in some periods [27].”

2) Authors have identified two tracks for testing: healthcare track/community track. It would be interesting to report results stratified by tracks, to see the weight of each track in the overall testing policy

Response: Thank you for your suggestion. As described in the manuscript, the proportion of PCR-test performed in the healthcare track was only 16.6%. In the supplementary material, we included the results of a sub-analysis including only PCR-tests from the community track, which showed the same associations as the main analysis. However, the older age groups were PCR-tested less in the community track compared to when the community and the healthcare track was analysed as a group (S3 Table).

3) Is there any ways to combine the database of PCR and Rapid antigen testing? I would interesting to have all analyses pooled, esp:
- How many people did not have neither a PCR nor a Rapid antigen test
- What is the proportion, at individual level, of test being done by PCR and Rapid antigen, if those who had rapid antigen also had PCR to confirm the diagnosis. In the latter case, if there were discrepancies.
- Authors barely mentioned the rate of positive PCR tests (only in a figure), it would be of interest to have a more detailed look

Response: Thank you for your valuable comment. During the epidemic, the PCR-test results were the primary basis for surveillance and national incidence figures. Moreover, PCR-tests have been primarily utilized in epidemiological studies. Individuals who tested positive by rapid antigen test were advised to have the infection confirmed in a subsequent PCR test. Therefore, we decided not to include rapid antigen-tests in all analyses. We added information about the proportion of individuals never PCR- or rapid antigen-tested (5.4%) to the manuscript (page 10). Lastly, we are not quite certain about what extra details of the positive PCR-tests the reviewer find relevant to include in the manuscript.

4) Discussion is too long, with comments that are not related to results being presented

Response: We thank the reviewer for this comment. However, we are not quite certain where precisely the reviewer suggests to shorten the Discussion. We feel, naturally, that the Discussion is of adequate length and that it covers the presented results. Also, this point had not previously been raised. Since decisions concerning the length of the manuscript also involve journal policy, we might suggest to keep the discussion at its present length and leave it to the editorial office, to decide if it should be rewritten.

5) The association between vaccination and testing should be more elaborated: those having > 2 doses may be person at risks (immmunocompromised), and thus be more tested in the context of their healthcare plan (hence the value of looking at the different testing tracks).

Response: We appreciate your comments and the time you have dedicated to review our work. In response to the specific point raised, we would like to address that individuals aged 18 years or above were offered 3 vaccine doses and the vaccination coverage in the total study population was above 60% (figure 1). Therefore, we do not believe that individuals having more than two doses are at higher risk or immunocompromised. We hope that you agree.
